# The Oral–Gut–Brain Axis in Pediatric Populations: The Implications of Oral Dysbiosis for Systemic Inflammation and Neuroinflammation

**DOI:** 10.3390/nu18152465

**Published:** 2026-07-29

**Authors:** Angelo Michele Inchingolo, Marco Severino, Grazia Marinelli, Lucia Casamassima, Paola Nardelli, Danilo Ciccarese, Andrea Palermo, Francesco Inchingolo, Alessio Danilo Inchingolo, Gianna Dipalma

**Affiliations:** 1 Department of Medicine and Surgery, University of Perugia, Severi Square n. 1, 06132 Perugia, Italy; angelomichele.inchingolo@unipg.it (A.M.I.); marco.severino@unipg.it (M.S.); 2 Department of Interdisciplinary Medicine, University of Bari “Aldo Moro”, 70124 Bari, Italy; grazia.marinelli@live.it (G.M.); lucia.casamassima@uniba.it (L.C.); paola.nardelli@uniba.it (P.N.); danilo.ciccarese@uniba.it (D.C.); alessiodanilo.inchingolo@uniba.it (A.D.I.); gianna.dipalma@uniba.it (G.D.); 3 Department of Experimental Medicine, University of Salento, 73100 Lecce, Italy

**Keywords:** oral–gut–brain axis, oral dysbiosis, pediatric microbiome, gut microbiota neuroinflammation, systemic inflammation, salivary cortisol, probiotics in children

## Abstract

**Background**: The oral microbiome plays a fundamental role in maintaining local and systemic health during childhood, a developmental period characterized by dynamic microbial, immune, and neuroendocrine maturation. Increasing evidence suggests that oral dysbiosis may influence gut microbiota composition, systemic inflammation, and neuroinflammatory pathways through the oral–gut–brain axis. **Aim**: This narrative review aimed to summarize and critically evaluate current evidence regarding the relationship between oral dysbiosis, gut microbial alterations, systemic inflammation, and neurodevelopmental processes in pediatric populations. **Methods**: A search of the literature was conducted using PubMed, Scopus, and Web of Science, including studies published between January 2016 and April 2026. Eligible studies included randomized controlled trials, observational studies, and reviews investigating at least one component of the oral–gut–brain axis in children or adolescents. **Results**: Current evidence supports a biological interaction between oral and gut microbiota through microbial translocation and immune-mediated mechanisms. Oral dysbiosis may contribute to gut microbial imbalance, intestinal barrier dysfunction, and systemic low-grade inflammation. Altered gut microbiota has been associated with neuroinflammatory signaling, hypothalamic–pituitary–adrenal (HPA) axis dysregulation, and adverse neurodevelopmental outcomes. Furthermore, pediatric randomized controlled trials suggest that probiotics and synbiotics can modulate oral and gut microbial composition, improve selected inflammatory and immune biomarkers, and reduce salivary cortisol levels. **Conclusions**: The oral–gut–brain axis represents a promising framework for understanding the systemic consequences of oral dysbiosis during childhood. However, direct evidence integrating oral, intestinal, immunological, and neurodevelopmental outcomes remains limited, highlighting the need for longitudinal and multidisciplinary pediatric studies.

## 1. Introduction

The oral microbiome represents one of the earliest microbial ecosystems established in humans and plays a fundamental role in maintaining both oral and systemic health during childhood [1,2,3]. Early microbial colonization is influenced by several prenatal and postnatal factors, including delivery mode, breastfeeding, antibiotic exposure, dietary habits, and oral hygiene practices [4,5]. During infancy and early childhood, microbial communities are highly dynamic and particularly susceptible to environmental perturbations that may alter microbial homeostasis [6,7,8].

In recent years, increasing attention has been directed toward the concept of the oral–gut–brain axis, a bidirectional network linking oral microbiota, intestinal microbial communities, systemic immune responses, and neurodevelopmental pathways [9,10,11]. For the purposes of this review, the oral–gut–brain axis is operationally defined as an integrated biological framework in which oral microbial communities may influence intestinal microbial ecology through microbial translocation and immune-mediated mechanisms, while gut microbial alterations may subsequently interact with the central nervous system through immune, endocrine, neural, and metabolic pathways [12,13]. Direct evidence supports the existence of bidirectional interactions between the oral and gut microbiota, as well as the role of gut microbiota in modulating immune and neuroendocrine signaling [14]. In contrast, the hypothesis that oral dysbiosis initiates a sequential cascade leading to gut dysbiosis, systemic inflammation, HPA-axis (hypothalamic–pituitary–adrenal) dysregulation, and neurodevelopmental alterations—particularly in pediatric populations—remains only partially supported by direct clinical evidence and is currently based on the integration of findings from pediatric, adult, and preclinical studies [15,16]. Accordingly, this review considers the oral–gut–brain axis as a biologically plausible conceptual model in which some individual components are supported by direct evidence, whereas the complete mechanistic pathway remains to be fully demonstrated, especially in children. Emerging evidence suggests that oral dysbiosis may extend beyond the oral cavity through microbial translocation and immune-mediated mechanisms, potentially influencing gut microbiota composition, intestinal permeability, systemic inflammation, and neuroinflammatory signaling [17,18,19,20,21]. While oral microbial translocation and gut dysbiosis have been documented in both pediatric and adult studies, the downstream effects on neuroinflammation and neurodevelopment are supported predominantly by adult and experimental evidence, with comparatively limited direct pediatric confirmation.

Although the gut–brain axis has been extensively investigated, the oral microbiome has only recently been considered a potential upstream contributor within this integrated framework [22]. In pediatric populations, this topic is particularly relevant because microbial ecosystems, immune maturation, and neurodevelopmental processes occur simultaneously during early life [23,24,25,26]. However, current evidence remains fragmented, and no comprehensive model has fully integrated oral dysbiosis, gut microbiota alterations, systemic inflammation, and neuroinflammatory pathways in children [10,22,27,28,29]. Consequently, the present review distinguishes evidence directly obtained from pediatric studies from mechanistic inferences extrapolated from adult and preclinical research when discussing the proposed oral–gut–brain axis.

In this context, microbiota-targeted nutritional strategies may represent a promising preventive approach in pediatric populations [30,31,32,33,34]. Nutrition also represents a major determinant of microbial development and immune maturation during early life [30,31,32,33,34,35,36,37,38]. Dietary patterns may significantly influence both oral and gut microbial ecosystems, modulating inflammatory pathways, intestinal barrier integrity, and neurodevelopmental processes [39,40,41,42,43,44,45,46,47,48,49]. In particular, Western dietary habits characterized by high sugar and ultra-processed food intake have been associated with microbial dysbiosis and chronic low-grade inflammation, whereas fiber-rich and Mediterranean dietary patterns may exert protective microbiota-mediated effects [50,51]. These observations further support the relevance of nutritional modulation within the framework of the oral–gut–brain axis [9,11,19].

The present study was conducted as a narrative review with the aim of summarizing and critically analyzing the available evidence regarding the relationship between oral dysbiosis, gut microbiota alterations, systemic inflammation, and neuroinflammatory mechanisms in pediatric populations, within the conceptual framework of the oral–gut–brain axis.

## 2. Materials and Methods

A comprehensive search of the literature was performed using the electronic databases PubMed, Scopus, and Web of Science (WoS), including studies published between January 2016 and April 2026. The literature search combined Medical Subject Headings (MeSH) terms and free-text keywords related to the oral microbiome (“oral microbiome”, “oral microbiota”, “oral dysbiosis”, “salivary microbiome”, “dental microbiome”), gut microbial alterations (“gut microbiota”, “gut microbiome”, “gut dysbiosis”, “oral–gut axis”, “oral–gut microbiota axis”), gut–brain communication (“gut–brain axis”, “microbiota–gut–brain axis”), neuroimmune and neuroendocrine pathways (“neuroinflammation”, “systemic inflammation”, “cortisol”, “salivary cortisol”, “HPA axis”), and pediatric populations (“children”, “child”, “pediatric”, “paediatric”, “infant”, “adolescent”, and “youth”). The complete search strategies for each database are reported in Appendix A.

### 2.1. Eligibility Criteria

Studies were considered eligible if they:

- Were published in English;
- Involved infants, children, or adolescents;
- Investigated at least one component of the oral–gut–brain axis;
- Evaluated oral microbiota, gut microbiota, systemic inflammation, neuroinflammation, HPA-axis activity, salivary cortisol, or microbiota-targeted interventions.

Randomized controlled trials, observational studies, narrative reviews, and systematic reviews were included.

Studies were excluded if they:

- Exclusively involved adult populations;
- Consisted of case reports, editorials, letters, or conference abstracts;
- Lacked relevance to the oral–gut–brain axis framework.

Although the primary focus of this review was pediatric clinical evidence, selected preclinical and animal studies were considered when providing mechanistic support for biological pathways related to microbial translocation, immune activation, gut–brain communication, and neuroinflammatory processes.

### 2.2. Study Selection and Data Extraction

The study selection process consisted of an initial screening of titles and abstracts, followed by full-text evaluation according to the predefined inclusion and exclusion criteria. Data extraction focused on study design, population characteristics, interventions, microbiological outcomes, inflammatory biomarkers, neurodevelopmental findings, and hormonal parameters.

### 2.3. Data Synthesis

Due to the heterogeneity of study designs, interventions, and outcome measures, a qualitative narrative synthesis approach was adopted. Particular emphasis was placed on randomized controlled trials, which were summarized separately to provide an overview of the highest level of available evidence in pediatric populations.

Overall, 278 records were initially identified. After duplicate removal and screening procedures, 13 pediatric randomized controlled trials were included in the final qualitative synthesis and summarized in Table 1. The findings were subsequently organized according to the major domains of the oral–gut–brain axis, including oral dysbiosis, oral–gut microbiota interactions, systemic inflammation, neuroinflammatory pathways, HPA-axis regulation, and microbiota-targeted interventions.

## 3. Oral Dysbiosis as an Upstream Factor

### 3.1. Development of the Oral Microbiome in Early Life

The oral microbiome begins to develop immediately after birth and undergoes rapid ecological succession throughout infancy and childhood [26,48]. Initial colonization patterns are strongly influenced by delivery mode, feeding practices, environmental exposure, and antibiotic administration [32,35,52]. Vaginal delivery promotes the acquisition of maternal vaginal and oral microbial communities, whereas cesarean delivery is associated with delayed microbial maturation and altered bacterial composition [8,53].

Breastfeeding contributes significantly to microbial homeostasis by providing bioactive compounds, immunological factors, and beneficial microorganisms that support microbial diversity and immune maturation [54,55,56,57]. Conversely, formula feeding has been associated with reduced microbial diversity and distinct microbial profiles. Additional factors, including oral hygiene habits, dietary sugar intake, and tooth eruption, further shape the composition and stability of oral microbial communities [33,36,37].

Because microbial ecosystems are still developing during childhood, the pediatric oral microbiome is particularly vulnerable to ecological perturbations [5,6,58,59]. These early-life alterations may have implications not only for oral diseases but also for systemic health outcomes [6,7,26,35,58].

### 3.2. Oral Dysbiosis and Pediatric Oral Diseases

Oral dysbiosis is characterized by an ecological imbalance within the oral biofilm involving reduced microbial diversity and the predominance of acidogenic and pro-inflammatory species [47,60,61,62,63,64]. In pediatric populations, dysbiosis is strongly associated with early childhood caries (ECC), one of the most prevalent chronic diseases worldwide [51,65,66,67,68].

The dysbiotic process involves increased abundance of cariogenic microorganisms such as *Streptococcus mutans* and *Lactobacillus* species, leading to acidification of the oral environment and enamel demineralization [69,70,71,72]. In addition to dental caries, dysbiosis contributes to gingival inflammation and altered host–microbe interactions [73,74,75]. These findings support the transition from a pathogen-centered concept of oral disease toward an ecological model based on microbial imbalance and immune dysregulation [58,76,77,78,79,80].

### 3.3. Evidence Supporting the Oral–Gut Axis

The oral cavity represents a major source of microorganisms entering the gastrointestinal tract through saliva. Approximately 10^11^ oral bacteria are swallowed daily, supporting the concept of biological continuity between oral and intestinal microbial ecosystems [2,12,81,82].

Several studies have demonstrated the presence of shared bacterial taxa between oral and fecal microbiota, including *Streptococcus*, *Veillonella*, *Prevotella*, and *Actinomyces species* [83,84]. Under physiological conditions, gastric acidity and intestinal barriers limit oral bacterial colonization of the gut [85]. However, in dysbiotic conditions, oral microorganisms may persist within the intestinal environment and contribute to microbial imbalance, epithelial barrier dysfunction, and immune activation [78,80,86].

This biological plausibility is further supported by preclinical evidence. In mouse models, oral administration of *Porphyromonas gingivalis* induced alterations in gut microbial composition, reduced expression of intestinal tight-junction proteins, increased endotoxemia, and promoted bacterial dissemination to distant organs, supporting the concept of microbial translocation along the oral–gut axis [87]. Similarly, Arimatsu et al. demonstrated that oral exposure to periodontal pathogens induced gut dysbiosis and systemic inflammatory responses in mice, further reinforcing the existence of a functional oral–gut connection [88].

This oral–gut interaction is increasingly recognized as a relevant pathway linking oral diseases with systemic inflammatory conditions [3,10,19,79,89]. In pediatric populations, where microbial and immune systems are still developing, oral dysbiosis may represent an early upstream factor capable of influencing gut microbial maturation and systemic immune responses [35,39,86].

### 3.4. Clinical Evidence from Pediatric RCTs (Randomized Controlled Trials)

Several pediatric randomized controlled trials have evaluated microbiota-targeted interventions for oral-health outcomes. In preschool children, probiotic milk containing *Lactobacillus rhamnosus SP1* was associated with lower caries prevalence and fewer new cavitated lesions compared with standard milk [90]. In a multicenter trial, *Lactobacillus paracasei* supplementation was associated with reduced caries risk and greater regression of carious lesions [91].

In children with early childhood caries, probiotic milk containing *Lactobacillus paracasei* was associated with increased salivary human neutrophil peptide 1–3 levels, reduced salivary *Streptococcus mutans*, and lower caries progression [92]. Other trials reported reductions in salivary S. mutans after supplementation with *Streptococcus salivarius M18* [93], *Lacticaseibacillus rhamnosus SD11* [71], or a probiotic mixture containing *Bifidobacterium* species [94].

Overall, these trials suggest that selected microbiota-targeted interventions may improve local oral outcomes, including caries-related measures, salivary bacterial counts, and selected salivary immune markers. However, the available pediatric RCTs do not directly demonstrate that oral microbiota modulation reduces systemic inflammation or modifies the complete oral–gut–brain axis.

## 4. From Gut Microbiota to Systemic Inflammation

### 4.1. Gut Dysbiosis and Barrier Dysfunction

Gut microbiota plays a central role in maintaining intestinal homeostasis, nutrient metabolism, epithelial barrier integrity, and immune regulation [5,41,95]. Alterations in microbial diversity and composition, commonly referred to as gut dysbiosis, may disrupt these physiological processes and contribute to increased intestinal permeability [78,96,97].

Compromised intestinal barrier function facilitates microbial translocation and exposure of the immune system to bacterial antigens and endotoxins [12,18]. This process promotes activation of innate and adaptive immune pathways and contributes to chronic low-grade systemic inflammation [3,17,98].

The transition from gut dysbiosis to systemic inflammation is mediated by several interconnected biological mechanisms. Reduced microbial diversity and depletion of beneficial commensals may impair short-chain fatty acid (SCFA) production, weaken epithelial tight junctions, and increase intestinal permeability [99,100]. This facilitates the translocation of bacterial components such as lipopolysaccharides into the systemic circulation, promoting activation of innate immune receptors and sustained cytokine production [101,102]. Rather than representing independent phenomena, gut dysbiosis, barrier dysfunction, and immune activation should be viewed as sequential stages of the same pathogenic process linking microbial imbalance to systemic inflammatory responses [5,41].

In pediatric populations, the consequences of gut dysbiosis may be particularly significant because intestinal and immune maturation occur simultaneously during early life [7,58,86,103]. Early microbial disturbances may therefore have long-term implications for immune regulation and susceptibility to inflammatory disorders [12,58,104].

### 4.2. Immune Activation and Systemic Inflammation

Gut dysbiosis is associated with increased production of pro-inflammatory cytokines, including interleukin-1β, interleukin-6, and tumor necrosis factor-α [38,105,106,107,108,109]. Altered microbial metabolites and impaired barrier integrity contribute to immune dysregulation and persistent inflammatory signaling [78,97].

Several mechanisms may explain the connection between oral dysbiosis and systemic inflammation [3,10,19,27,82]. Oral pathogens and inflammatory mediators may influence gut microbial composition directly through microbial translocation or indirectly through immune activation [10,21]. This interaction reinforces the concept of a continuous oral–gut inflammatory axis [9,17,18,22].

Systemic low-grade inflammation has been associated with several pediatric conditions, including metabolic disturbances, immune-mediated diseases, and neurodevelopmental alterations [22,110]. Consequently, microbial dysbiosis may represent a shared biological pathway linking oral health and systemic disease [10,27,82,84].

### 4.3. Extracellular Vesicles as Emerging Mediators of Oral–Gut Communication

In addition to the translocation of whole microorganisms through saliva, increasing evidence suggests that extracellular vesicles (EVs) represent an important mechanism of communication within the oral–gut–brain axis. Both host-derived extracellular vesicles and bacterial outer membrane vesicles (OMVs) can transport bioactive cargo, including proteins, lipids, nucleic acids, metabolites, and virulence factors, thereby mediating local and systemic intercellular signaling. These vesicles are increasingly recognized as key mediators of host–microbiome crosstalk and immune regulation [110,111].

The oral cavity represents a major source of extracellular vesicles because saliva contains abundant host-derived EVs released by epithelial, immune, and salivary gland cells, together with bacterial OMVs produced by both commensal and pathogenic microorganisms. Periodontal pathogens, including *Porphyromonas gingivalis*, are able to release OMVs enriched in lipopolysaccharides, gingipains, proteases, and other immunomodulatory molecules that may disseminate beyond the oral cavity and amplify systemic inflammatory responses. Compared with whole bacterial translocation, vesicle-mediated transport may facilitate the delivery of microbial products to distant tissues while avoiding direct bacterial colonization [110].

Recent evidence further suggests that extracellular vesicles may participate in oral–gut communication by influencing intestinal epithelial integrity, immune activation, and microbial ecological balance. Through these mechanisms, EVs may contribute to the biological continuity between oral dysbiosis and gut dysbiosis already discussed in this review. Furthermore, EV-associated microbial molecules may indirectly influence neuroimmune signaling by promoting systemic cytokine production and HPA-axis activation. Although these mechanisms are biologically plausible, direct pediatric evidence remains limited, and current knowledge is largely derived from experimental models and adult studies [111]. Therefore, extracellular vesicles should currently be regarded as an emerging mechanistic component of the proposed oral–gut–brain axis rather than a clinically established pathway in children [110,111,112,113,114].

### 4.4. Clinical Evidence from Pediatric RCTs

Randomized controlled trials investigating gut microbiota-targeted interventions in children demonstrated that probiotics may influence gut microbial composition, mucosal immunity, and intestinal homeostasis [39,66,94,105]. Supplementation with *Lactobacillus paracasei N1115* was associated with increased fecal *Lactobacillus* abundance, maintenance of fecal pH, and enhancement of secretory IgA levels in young children [30,91,115].

Additional studies reported beneficial modulation of gut microbial development and improved tolerability following probiotic administration during infancy and early childhood [5,36,64,116]. Although the included trials varied considerably in probiotic strains, dosages, and outcomes, they collectively support the biological relevance of microbiota modulation in pediatric systemic health [39,45,94,117,118].

## 5. The Gut–Brain Axis and Neuroinflammatory Pathways

### 5.1. Neural Communication and the Vagus Nerve

The gut–brain axis is a bidirectional communication system connecting the gastrointestinal tract and the central nervous system through neural, endocrine, immune, and metabolic pathways [24,78,98,117,118]. Among these mechanisms, vagal signaling represents one of the principal routes of microbiota–brain communication [9,11,19,25,47].

The vagus nerve transmits signals from intestinal microbial activity to the brain and modulates autonomic and inflammatory responses [47]. Evidence supporting this mechanism derives primarily from animal studies. In a landmark mouse experiment, administration of *Lactobacillus rhamnosus* JB-1 altered emotional behavior and central GABA receptor expression through a vagus-dependent pathway, whereas these effects disappeared following vagotomy, demonstrating the critical role of vagal signaling in microbiota–brain communication [119]. Microbial metabolites and inflammatory mediators may influence vagal afferent pathways, thereby affecting stress responses, emotional regulation, and neurodevelopmental processes [25,120,121].

### 5.2. Cytokines and Immune-Mediated Neuroinflammation

Systemic inflammation induced by microbial dysbiosis may contribute to neuroinflammatory signaling through circulating cytokines and immune mediators [29,79,122]. Elevated levels of pro-inflammatory cytokines have been associated with altered blood–brain barrier permeability, microglial activation, and impaired neuronal signaling [24,106,123]. Preclinical studies also support the link between oral dysbiosis and neuroinflammation. In mice, chronic oral exposure to *P. gingivalis* resulted in the disruption of intestinal barrier integrity, activation of IL-17A-mediated inflammatory pathways, microglial activation, and neuroinflammatory changes within the central nervous system, providing mechanistic evidence connecting oral microbial imbalance with brain inflammatory responses [124,125,126,127,128].

These mechanisms are particularly relevant during childhood because neurodevelopmental processes remain highly sensitive to inflammatory stimuli [24,25,47,70]. Chronic low-grade inflammation may therefore influence cognitive function, emotional regulation, and neurodevelopmental trajectories [24,25,47,70,117,120].

### 5.3. Short-Chain Fatty Acids and Microbial Metabolites

SCFAs, including acetate, propionate, and butyrate, are among the most important microbial metabolites involved in gut–brain communication [25,38,47,60,63]. SCFAs contribute to intestinal barrier integrity, modulation of immune responses, and regulation of neurotransmitter pathways [24,25,38,47,63].

Altered SCFA production during dysbiosis may influence neuroinflammatory pathways and brain function [25,38,47,63]. Experimental and clinical evidence suggests that microbial metabolites can affect neuroplasticity, stress regulation, and behavioral outcomes [23,25,121].

### 5.4. Neurodevelopmental Implications in Pediatric Populations

Increasing evidence supports a relationship between gut microbiota composition and neurodevelopmental outcomes in children [24,25,47,49,63,118]. Dysbiosis has been associated with cognitive dysfunction, behavioral alterations, anxiety, depression, and neurodevelopmental disorders [11,47,118,125].

Pediatric randomized controlled trials further support this interaction. In adolescents with major depressive disorder, probiotic supplementation combined with standard therapy improved cognitive performance and reduced inflammatory markers such as IL-1β and cortisol levels [11,105,125]. Another trial involving extremely preterm infants reported improved language outcomes following probiotic supplementation, although no significant effects on global neurodevelopment were observed [70].

Although causality remains difficult to establish, current evidence suggests that microbiota modulation may influence neurodevelopmental and neuroinflammatory processes during childhood [24,25,47,70].

Importantly, these communication pathways should not be considered independent mechanisms. Microbial metabolites, cytokine signaling, vagal activation, and neuroendocrine responses operate as an integrated network through which intestinal microbial alterations may influence brain function. Dysbiosis-induced inflammation can modify blood–brain barrier permeability, facilitate microglial activation, and alter neurotransmitter synthesis, while changes in microbial metabolite production may simultaneously affect neuronal plasticity and stress responsiveness. This integrated model provides a mechanistic explanation for the observed associations between microbial imbalance and neurodevelopmental outcomes reported in pediatric studies.

## 6. The HPA Axis and Salivary Cortisol as an Integrative Biomarker

### 6.1. Interactions Between the Microbiota and the HPA Axis

The HPA axis is one of the primary neuroendocrine systems regulating stress responses and homeostasis [9,110,121]. Emerging evidence indicates that the gut microbiota can modulate HPA-axis activity through immune, neural, and metabolic pathways [9,103,125,126,127]. Mechanistic support for this concept comes from germ-free animal models. Sudo et al. demonstrated that germ-free mice exhibited exaggerated HPA-axis responses to stress compared with conventionally colonized animals and that microbial colonization during early life partially normalized this response. These findings suggest that microbiota composition contributes to the developmental programming of neuroendocrine stress regulation [129].

Microbial dysbiosis may contribute to altered cortisol secretion through activation of inflammatory pathways and vagal signaling [105]. Chronic stress and elevated cortisol levels may negatively affect microbial diversity and intestinal barrier function, reinforcing a bidirectional microbiota–stress interaction [18,22,24,78,97,106,123].

### 6.2. Salivary Cortisol in Pediatric Research

Salivary cortisol is widely used as a non-invasive biomarker of HPA-axis activity in pediatric populations [7,30,79,115]. Compared with serum measurements, salivary sampling is particularly suitable for children because it is simple, painless, and stress-free [130,131].

In recent years, salivary cortisol has emerged as a potential integrative biomarker linking microbial dysbiosis, systemic inflammation, and stress responses [120]. Variations in cortisol levels may therefore reflect broader neuroendocrine and inflammatory alterations associated with the oral–gut–brain axis [78,79,118].

However, the interpretation of salivary cortisol as a stable integrative biomarker requires important methodological caution. Cortisol secretion is strongly influenced by multiple confounding factors, including circadian rhythm (diurnal variation with peak levels in the early morning), acute psychological stress [132,133], sleep quality and duration, recent food intake and dietary composition, physical activity [134,135], pharmacological treatments (e.g., corticosteroids, psychotropic medications), and the timing and standardization of sample collection procedures [132,134]. Variability in saliva sampling protocols (e.g., passive drool vs. swab methods), handling, and storage conditions may further affect measured concentrations [133,136]. These sources of variability can significantly influence cortisol levels independently of microbiota-related mechanisms and must be carefully controlled or at least acknowledged when interpreting findings in pediatric studies [132,133]. Therefore, salivary cortisol should be considered a sensitive but non-specific marker of HPA-axis activity rather than a direct or exclusive proxy of microbiota–brain interactions.

### 6.3. Evidence from Microbiota-Targeted Interventions

Evidence from pediatric intervention studies examining cortisol-related outcomes is limited and heterogeneous. In a semi-randomized controlled study involving school-aged children, daily consumption of a probiotic fermented dairy product was associated with lower salivary cortisol concentrations than placebo or diet-as-usual interventions [120].

In a randomized, triple-blind, placebo-controlled trial in cesarean-delivered infants and toddlers, supplementation with Lacticaseibacillus paracasei N1115 was associated with a reduction in salivary cortisol from baseline, maintenance of fecal pH, and increased fecal Lactobacillus abundance [115].

In adolescents with major depressive disorder, probiotic supplementation combined with sertraline was associated with lower serum cortisol and interleukin-1β concentrations compared with sertraline alone [105].

Collectively, these studies suggest that microbiota-targeted interventions may influence cortisol-related outcomes in selected pediatric settings. However, differences in study design, population, probiotic formulation, co-interventions, and cortisol matrix prevent direct comparison across studies and do not establish that these effects are mediated by modulation of the complete oral–gut–brain axis [105,115,120].

## 7. The Role of Diet in Modulating the Oral–Gut–Brain Axis

### 7.1. Early-Life Nutrition and Microbiota Development

Nutrition represents one of the most important modulators of microbial development during infancy and childhood [36,37,48,137]. Breastfeeding promotes microbial diversity and immune maturation through the delivery of bioactive compounds, immunoglobulins, and beneficial microorganisms. In contrast, formula feeding may alter microbial composition and reduce microbial diversity [25,35,42,137].

Complementary feeding practices also contribute to microbial maturation and metabolic programming [138]. Dietary exposures during early life may therefore influence oral and gut microbiota composition with long-term consequences for immune and neurodevelopmental health [6,29,36]. Early nutritional programming is particularly relevant because it occurs during a critical window of immune, endocrine, and microbiome co-development, in which dietary exposures may shape host–microbe interactions with long-term systemic consequences.

### 7.2. Oral Microbial Ecology and Dietary Drivers

Dietary habits strongly influence oral microbial ecology [6]. Nutrient composition, food consistency, and dietary frequency all contribute to shaping the oral ecosystem, particularly through their effects on substrate availability for microbial metabolism. Instead, diets characterized by high intake of refined carbohydrates and industrially processed foods can favor ecological shifts toward acidogenic and aciduric microbial communities, thereby increasing caries susceptibility [2,4,28,29,45,48].

Conversely, balanced dietary patterns rich in fiber, micronutrients, and anti-inflammatory compounds may support microbial homeostasis and oral health [128]. Nutritional interventions may therefore represent an important strategy for preventing dysbiosis-related oral diseases during childhood. In addition, dietary habits may influence salivary composition, buffering capacity, and local immune responses, further affecting microbial homeostasis within the oral cavity [66,72,90,91,94]. Emerging evidence also suggests that micronutrient deficiencies, including inadequate intake of vitamin D, calcium, iron, and zinc, may impair mucosal immunity and increase susceptibility to dysbiosis-related oral diseases [40,61,139,140]. Dietary composition may also indirectly influence oral ecological stability by modulating salivary properties, buffering capacity, and local immune responses. Micronutrient availability (e.g., vitamin D, calcium, iron, zinc) may further contribute to mucosal immune competence and resistance to dysbiosis.

### 7.3. Dietary Modulation of Gut Inflammation and Neurodevelopment

Diet also plays a key role in regulating gut microbial composition and inflammatory responses. Fiber-rich diets promote the production of SCFAs, which exert anti-inflammatory and neuroprotective effects [141,142]. SCFAs such as butyrate, acetate, and propionate represent key metabolic mediators linking dietary intake to host immune regulation, epithelial integrity, and neuroimmune signaling pathways.

Dietary patterns such as the Mediterranean diet have been associated with improved microbial diversity and reduced systemic inflammation [143,144,145,146,147]. Additionally, probiotics and prebiotics may contribute to the modulation of both oral and gut microbial ecosystems [36,41,117,118].

**Figure 1:**
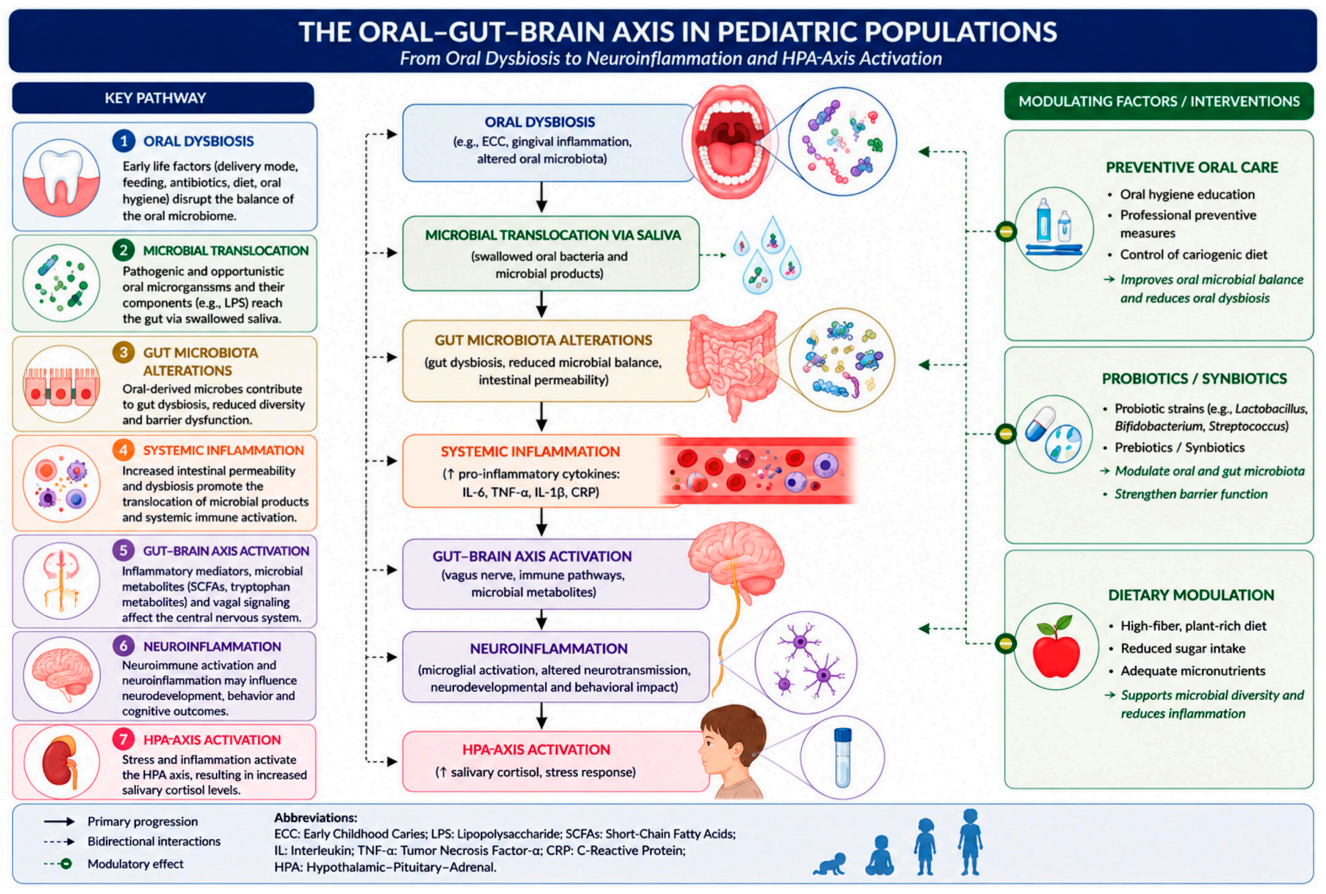
A conceptual representation of the oral–gut–brain axis in pediatric populations. The figure illustrates the potential biological pathway linking oral dysbiosis to gut microbiota alterations, systemic inflammation, neuroinflammation, and HPA-axis activation in children. It also highlights the potential modulatory role of probiotics, preventive oral care, and dietary interventions.

Taken together, these findings suggest that dietary strategies may represent a relevant therapeutic approach for modulating the oral–gut–brain axis in pediatric populations (Figure 1) [10,24,25,27,43,47,49,77].

### 7.4. Microbiota-Targeted Interventions in Pediatric Populations

Several randomized controlled trials have investigated the effects of probiotics, synbiotics, and related microbiota-targeted interventions on oral health, gut microbiota composition, inflammatory markers, stress-related biomarkers, and neurodevelopmental outcomes in pediatric populations [70,91,105,115]. Collectively, these studies suggest that modulation of the microbiota may contribute to improvements in oral microbial balance, reductions in cariogenic bacteria, enhancement of selected immune markers, modulation of salivary cortisol levels, and, in some cases, favorable neurodevelopmental outcomes [70,92,115,120]. A structured summary of the pediatric randomized controlled trials included in this review is presented in Table 1.

## 8. Discussion

This narrative review proposes a conceptual framework in which oral dysbiosis may represent an upstream contributor to gut microbial alterations, systemic inflammation, and stress-related neuroendocrine dysregulation during childhood.

Experimental studies indicate that oral administration of *Porphyromonas gingivalis* can alter gut microbial composition, impair intestinal barrier function, and promote systemic inflammatory changes [87,88].

Separate experimental and mechanistic evidence indicates that gut microbiota may influence neuroendocrine stress responses through immune, neural, and metabolic pathways, although much of this evidence derives from animal models [13,129].

However, direct pediatric evidence connecting oral dysbiosis, gut dysbiosis, systemic inflammation, HPA-axis dysregulation, and neurodevelopmental outcomes within a single causal pathway remains limited. Available pediatric intervention studies have assessed selected peripheral outcomes, such as salivary cortisol, fecal microbial markers, and immune biomarkers, rather than the complete oral–gut–brain cascade [115,120].

Although this conceptual model is supported by biological evidence, direct evidence demonstrating the complete oral–gut–brain axis in pediatric populations remains limited, and several mechanistic aspects are extrapolated from adult and preclinical studies.

Unlike prior reviews that have addressed individual components of oral dysbiosis, oral–gut interactions, or the microbiota–gut–brain axis, the present review specifically integrates these domains within a pediatric developmental framework, while distinguishing direct pediatric evidence from findings extrapolated from adult and preclinical studies [148,149,150].

By synthesizing evidence from oral health, microbiome research, immunology, neurodevelopment, and microbiota-targeted interventions, this review proposes a developmental model in which oral dysbiosis may represent a potential upstream contributor to systemic and neuroinflammatory processes during childhood [151,152].

This perspective is particularly relevant because childhood is characterized by the simultaneous maturation of the microbiota, immune system, endocrine pathways, and central nervous system, making these biological systems especially susceptible to environmental and microbial influences [24,25,26,48]. However, while these developmental characteristics are well established in pediatric populations, the mechanistic interactions linking the oral microbiota, gut microbiota, and brain have only been partially demonstrated in children and are largely inferred from experimental and adult evidence.

The available evidence supports the existence of biological continuity between oral and intestinal microbial ecosystems [10,19,20,27,85]. This continuity has been documented in both pediatric and adult studies, whereas the downstream systemic and neurodevelopmental consequences are primarily supported by adult and preclinical evidence. Oral microorganisms may influence gut microbial composition through salivary translocation and immune-mediated pathways, particularly under dysbiotic conditions [10,14,21,83,153]. Most mechanistic evidence supporting these pathways originates from experimental models and adult populations, with only limited direct pediatric confirmation.

Gut dysbiosis, in turn, may contribute to increased intestinal permeability, immune activation, and chronic low-grade inflammation [26,40,78,153]. These interconnected processes provide a biologically plausible mechanism through which oral microbial imbalance may extend beyond the oral cavity and contribute to systemic inflammatory responses. Nevertheless, in pediatric populations, this integrated pathway remains biologically plausible rather than conclusively demonstrated.

Emerging evidence further suggests that inflammatory mediators, microbial metabolites, vagal signaling, and HPA-axis activation collectively link gut microbial alterations to neurodevelopmental and neurobehavioral outcomes [24,25,47,127].

Within this framework, one of the most important findings emerging from the available evidence is the growing body of literature on microbiota-targeted interventions in pediatric populations. The randomized controlled trials summarized in Table 1 indicate that selected probiotic interventions may influence oral-health outcomes, including caries-related measures and salivary immune markers [91,92].

In other pediatric settings, *Lacticaseibacillus paracasei N1115* supplementation was associated with changes in fecal *Lactobacillus* abundance and maintenance of fecal pH, whereas a probiotic fermented dairy intervention was associated with lower salivary cortisol concentrations in school-aged children [115,120].

Evidence concerning neurodevelopmental outcomes remains preliminary. In extremely preterm infants, *Limosilactobacillus reuteri* supplementation was associated with higher language scores at two years, although no significant differences were observed in overall neurodevelopment or growth [70]. In adolescents with major depressive disorder, probiotic supplementation combined with sertraline was associated with improved cognitive performance and lower cortisol and IL-1β concentrations [105].

These findings provide direct pediatric evidence for selected oral, intestinal, immune, and neuroendocrine outcomes relevant to the proposed oral–gut–brain framework.

It is important to explicitly emphasize that, although randomized controlled trials in pediatric populations provide evidence that probiotic interventions can modulate oral and gut microbial composition and influence selected immune and salivary biomarkers, they do not directly demonstrate causal modulation of the complete oral–gut–brain axis, particularly with respect to neuroinflammation, central nervous system signaling, or long-term neurodevelopmental outcomes. Therefore, the interpretation of these findings should remain limited to microbiota modulation and associated peripheral biomarkers, avoiding any overextension toward conclusions regarding full axis integration.

Despite these promising findings, the interpretation of current clinical evidence requires caution. Considerable heterogeneity exists regarding probiotic strains, dosage regimens, intervention duration, participant characteristics, and outcome measures. Most studies have focused on oral-health outcomes, particularly caries prevention and reductions in Streptococcus mutans colonization, whereas relatively few have simultaneously evaluated gut microbiota composition, inflammatory biomarkers, neurodevelopmental outcomes, and HPA-axis activity. Consequently, direct pediatric evidence supporting a fully integrated oral–gut–brain mechanism remains limited, and many mechanistic interpretations continue to rely on evidence extrapolated from adult and experimental studies.

An important aspect emerging from the current literature concerns the distinction between probiotics, prebiotics, postbiotics, and broader nutritional approaches. To date, probiotics represent the intervention supported by the strongest pediatric clinical evidence, whereas studies investigating prebiotics and postbiotics remain comparatively limited [30,70,94,105]. Prebiotics may exert beneficial effects indirectly by stimulating the growth and metabolic activity of endogenous beneficial microorganisms, thereby increasing SCFA production and supporting intestinal barrier integrity [36,41,42]. Most evidence supporting these mechanisms derives from adult and preclinical studies. Postbiotics, including microbial metabolites and inactivated microbial products, have recently attracted interest because they may provide immunomodulatory benefits while avoiding some of the safety and stability concerns associated with live microorganisms [40,46]. However, pediatric clinical evidence evaluating postbiotics remains scarce. Although these approaches are promising, robust pediatric clinical trials directly comparing their efficacy are currently lacking.

Nutritional modulation represents another particularly attractive strategy because it may simultaneously influence multiple components of the oral–gut–brain axis. Dietary habits affect both oral and intestinal microbial ecosystems and play a fundamental role in immune maturation and inflammatory regulation [32,33,35,36,37,38,39,43,50,51]. Evidence supporting these effects on oral and gut microbiota is available in pediatric populations, whereas their downstream neurodevelopmental implications are supported predominantly by adult and experimental evidence. Diets rich in fiber, plant-derived nutrients, and anti-inflammatory compounds may support microbial diversity, promote SCFA production, and reduce systemic inflammatory burden [43,50,51,71]. In contrast, Western dietary patterns characterized by excessive consumption of refined sugars and ultra-processed foods may contribute to oral dysbiosis, gut microbial imbalance, and chronic low-grade inflammation [50,51]. From a translational perspective, nutritional interventions may offer broader and more sustainable ecosystem-level effects than single-strain supplementation because they simultaneously influence both oral and gut microbial communities.

The potential clinical implications of these findings extend beyond traditional oral-health outcomes. If future pediatric longitudinal studies confirm the causal relationships proposed within the oral–gut–brain axis, oral dysbiosis could become an early and potentially modifiable biomarker of broader systemic risk [17,18,19,20,21,22,27]. Preventive strategies combining oral hygiene promotion, dietary optimization, and microbiota-targeted interventions may contribute not only to reducing dental disease but also to supporting immune regulation, stress resilience, and neurodevelopmental health [24,25,27,39,46]. However, these broader systemic benefits remain biologically plausible and require confirmation through direct pediatric evidence integrating oral, gut, immune, endocrine, and neurodevelopmental outcomes. Such an approach would align with current trends toward preventive and personalized pediatric medicine.

## 9. Evidence Gaps, Limitations and Future Directions

Despite the increasing evidence linking oral microbiota, gut microbiota, immune function, and neuroendocrine regulation in pediatric populations, important gaps in knowledge still limit a full mechanistic and clinical understanding of the oral–gut–brain axis [9,10,11,17]. Current data are largely fragmented across different biological domains, study designs, and outcome measures, making it difficult to establish temporally and causally coherent relationships. For this reason, future research should move toward more integrative and methodologically standardized approaches [19,20,22,43]. A key priority is the development of longitudinal pediatric cohort studies that investigate, within the same population and over time, multiple interconnected layers of the proposed axis. In particular, future studies should concurrently assess oral microbiome composition and dynamics, gut microbiome composition and dynamics, dietary patterns and nutritional exposures, salivary cortisol as a marker of HPA-axis activity, systemic and local inflammatory biomarkers including cytokines and acute-phase mediators, extracellular vesicle (EV) profiling as potential mediators of host–microbe communication, and neurodevelopmental outcomes encompassing cognitive, behavioral, and emotional domains [110,111,112,113,114,115,116]. The integration of these domains within a single cohort is essential to move beyond correlative findings and to determine whether oral dysbiosis plays a causal upstream role or represents only a parallel manifestation of broader systemic alterations. In addition, future research should carefully address methodological standardization, particularly for variables that are highly sensitive to external influences. This is especially relevant for salivary cortisol, which is strongly affected by circadian rhythm, acute stress, sleep quality, dietary intake, medication use, and sampling procedures. Standardized protocols for timing of collection and pre-analytical handling will therefore be essential to ensure comparability across studies and to reduce confounding bias [19,20,22]. Similar standardization is also required for microbiome sequencing methods, inflammatory biomarker assessment, and neurodevelopmental evaluation tools, given the substantial heterogeneity currently observed in the literature. Advanced multiomics approaches integrating metagenomics, metabolomics, proteomics, and extracellular vesicle profiling may further contribute to a more functional and mechanistic understanding of host–microbe interactions, moving beyond descriptive taxonomic shifts toward biologically meaningful pathways [79,90,104,124]. Such approaches may help clarify how microbial ecosystems interact with host immune and neuroendocrine systems during critical windows of pediatric development. Finally, well-designed longitudinal interventional studies are needed to determine whether microbiota-targeted strategies, including probiotics, dietary interventions, or combined approaches, can exert sustained effects not only on oral and gut microbial ecosystems but also on systemic inflammation, stress regulation, and neurodevelopmental trajectories. Addressing these gaps will be essential to transition from an associative and partially inferred evidence base toward a fully validated and clinically actionable model of the pediatric oral–gut–brain axis [9,10,11,17].

Despite these promising findings, several limitations of the current evidence should be acknowledged. Most studies remain relatively small, involve short follow-up periods, and employ heterogeneous methodologies that limit comparability [30,66,70,71,90,91,92,93,94,105,115,120,153]. In addition, many investigations rely on surrogate biomarkers rather than clinically meaningful long-term outcomes. The absence of standardized microbiome assessment methods and the limited integration of oral, intestinal, immunological, hormonal, and neurodevelopmental measures represent important methodological challenges [19,22,25,27,47].

Furthermore, direct mechanistic evidence in pediatric populations remains scarce. Consequently, some of the biological pathways discussed in this review, including microbial translocation, vagal signaling, HPA-axis modulation, and neuroinflammatory activation, are partly supported by experimental animal studies. These preclinical data provide biological plausibility but should not be interpreted as direct evidence of causality in children.

Future research should prioritize longitudinal pediatric cohorts, standardized microbiome characterization protocols, and integrated multiomics approaches capable of simultaneously evaluating oral microbiota, gut microbial ecology, inflammatory mediators, neurodevelopmental trajectories, and HPA-axis activity [17,19,22,24,25,27,47,48]. Such studies will be essential for determining whether microbiota-targeted interventions can truly modify the biological pathways linking oral dysbiosis to systemic inflammation and neurodevelopmental health.

## 10. Conclusions

The oral–gut–brain axis represents a biologically plausible and clinically relevant framework linking oral dysbiosis, gut microbial alterations, systemic inflammation, neuroinflammatory signaling, and neuroendocrine regulation in pediatric populations.

Current evidence suggests that microbiota-targeted interventions may influence oral and intestinal microbial communities, inflammatory pathways, and stress-related biomarkers such as salivary cortisol. However, direct evidence integrating all components of the oral–gut–brain axis remains limited.

Future research should focus on longitudinal pediatric studies, standardized methodologies, and integrated multiomics approaches capable of simultaneously evaluating oral, intestinal, immunological, and neurodevelopmental outcomes.

A deeper understanding of the oral–gut–brain axis may open new perspectives for preventive and therapeutic strategies aimed at promoting both oral and systemic health from early life onward.

## Figures and Tables

**Table 1:** Pediatric randomized controlled trials evaluating probiotics, synbiotics, and related microbiota-targeted interventions.

Authors	Study Design	Participants	Axis Component	Material and Methods	Outcomes
Rodríguez et al., 2016 [90]	Cluster-randomized trial	Preschool children at high risk of caries	Oral	**Strain:** *Lactobacillus rhamnosus*; **Dose:** probiotic milk containing 10^7^ CFU/mL (Colony-Forming Units per milliliter); 150 mL administered on weekdays; **Duration:** 10 months; triple-blind, placebo-controlled randomized trial in preschool children aged 2–3 years attending nursery schools in Chile.	Probiotic group showed lower caries prevalence and significantly fewer new cavitated lesions compared with controls.
Piwat et al., 2020 [91]	Multicenter RCT	Preschool children	Oral	**Strain:** *Lactobacillus paracasei*; **Dose:** probiotic milk powder administered daily or 3 times/week; **Duration:** 6 months; double-blind randomized controlled trial in preschool children.	Both probiotic regimens reduced caries risk and significantly increased regression of carious lesions compared with placebo.
Wattanarat et al., 2021 [92]	Randomized controlled trial	Preschool children with ECC/severe ECC and caries-free group	Immune + Oral	**Strain:** *Lactobacillus paracasei*; **Dose:** probiotic milk once daily or 3 times/week; **Duration:** 6 months intervention with follow-up at 12 months; study in preschool children with ECC/severe-ECC.	Probiotic groups showed increased salivary HNP1-3 levels, reduced *Streptococcus mutans* levels, and decreased caries progression, especially in severe-ECC children.
Wang et al., 2021 [30]	Double-blind placebo-controlled RCT	6 mesi–3 anni (≥6 months and ≤3 years	Gut	**Strain:** *Lactobacillus paracasei* N1115; **Dose:** 2 g/day of probiotic supplement (~4.8 × 10^9^ CFU/day) vs. placebo; **Duration:** 8 weeks.	Well tolerated. No major differences vs. placebo in stool, symptoms, cortisol, or SCFAs; slight increase in *Lactobacillus* abundance in probiotic group at weeks 4 and 8.
Brett et al., 2021 [120]	Semi-randomized controlled trial	262 children aged 4–7 years	HPA-cortisol	**Strain:** not reported (probiotic fermented dairy food); **Dose:** daily consumption on school days; **Duration:** 1 school semester.	Probiotic fermented dairy vs. placebo/usual diet; lower salivary cortisol levels in the probiotic group, with no differences in salivary CRP (C-reactive Protein).
Staszczyk et al., 2022 [66]	Clinical trial	Pediatric population: 140 children aged 3–6 years	Oral	**Strain:** thermally inactivated *Lactobacillus salivarius*; **Dose:** chewing tablets daily for 2 weeks; **Duration:** 12-month follow-up.	Probiotic group showed significantly lower caries incidence and prevalence at 12 months vs. control.
Li et al., 2023 [115]	Randomized, triple-blind, placebo-controlled trial	109 cesarean-delivered infants/toddlers (6–24 months; 101 completed)	Integrated oral–gut–brain	**Strain:** *Lacticaseibacillus paracasei* N1115; **Dose:** 2 × 10^9^ CFU/g (una bustina al giorno, vs. placebo maltodestrina); **Duration:** 12 weeks.	Probiotic maintained fecal pH, reduced salivary cortisol, and increased fecal *Lactobacillus* (especially at week 4); some increase in fecal sIgA (secretory Immunoglobulin A) in 6–12 months subgroup.
Salim et al., 2023 [93]	Randomized clinical trial	40 preprimary children aged 3–6 years	Oral	**Strain:** *Streptococcus salivarius* M18 (BLIS M18 probiotic); **Dose:** 1 lozenge/day for 7 days (vs. unsweetened placebo lozenge); **Duration:** 1 week.	Probiotic group showed significant reduction in *Streptococcus mutans* counts, with increased buffering capacity and no significant change in salivary pH.
Teanpaisan et al., 2023/2024 [71]	Double-blind randomized controlled trial	121 adolescent subjects aged 13–14 years	Oral	**Strain:***Lacticaseibacillus rhamnosus* SD11; **Dose:** 1 lozenge/day containing 10^8^ CFU/g (vs placebo lozenge without strain); **Duration:** 8 weeks (probiotic administered for the first 4 weeks with follow-up to 8 weeks).	Probiotic group showed reduced *Streptococcus mutans* and increased total lactobacilli, with higher microbial diversity and no adverse effects.
Shi et al., 2023 [105]	Randomized controlled trial	180 adolescents aged 17–19 years with major depressive disorder (MDD)	Integrated oral–gut–brain	**Strain:** probiotic combination (*Bifidobacterium* spp., *Lactobacillus* spp., *Enterococcus* spp. and *Bacillus cereus*); **Dose:** 0.5 g twice daily (vs sertraline only control group); **Duration:** 2 months; randomized clinical trial in adolescents aged 17–19 years with major depressive disorder (MDD).	Probiotic group showed improved cognitive performance (RBANS scores) and reduced cortisol and IL-1β (Interleukin-1 beta) levels vs. control.
Olczak-Kowalczyk et al., 2024 [146]	Randomized controlled trial	73 children aged between 3 and 6 years to participate	Oral	**Strain:** inactivated *Ligilactobacillus salivarius* + cranberry extract; **Dose:** daily tablet (paraprobiotic) vs. placebo; **Duration:** 3-month intervention, 9-month follow-up.	Test group showed significantly lower incidence of initial carious lesions (ICDAS 1–2) at 9 months (*p* < 0.05); no changes in *S. mutans*, lactobacilli counts or plaque levels in either group.
Uyar et al., 2024 [94]	Randomized controlled clinical study	58 children aged 2–12 years	Oral	**Strain:** probiotic mixture containing *Bifidobacterium breve*, *Bifidobacterium infantis*, *Bifidobacterium longum*, and *Bifidobacterium bifidum*; **Dose:** 6 drops/day (≈5 × 10^8^ CFU per drop) vs. no probiotic (control); **Duration:** 6 months follow-up (probiotic administered for the first 3 months, then discontinued for 3 months).	Probiotic group showed significantly lower *Streptococcus mutans* levels at all follow-ups; no significant differences in Lactobacillus species between groups.
Wejryd et al., 2025 [70]	Randomized double-blind placebo-controlled trial (secondary analysis)	134 extremely preterm infants	Neurodevelopment	**Strain:** *Limosilactobacillus reuteri*; **Dose:** 2.5 × 10^8^ CFU daily enteral supplementation vs. placebo; **Duration:** from ≤72 h after birth to postmenstrual week 35–36; follow-up to 2 years corrected age.	No differences in overall growth or neurodevelopment, but language scores were significantly higher in the probiotic group at 2 years.

## Data Availability

No new data were created or analyzed in this study. Data sharing is not applicable to this article.

## Abbreviations

The following abbreviations are used in this manuscript:

CFU/mL	Colony-Forming Units Per Milliliter
CRP	C-reactive Protein
ECC	Early Childhood Caries
HNP1-3	Human Neutrophil Peptides 1–3
HPA	Hypothalamic–Pituitary–Adrenal
ICDAS	International Caries Detection and Assessment System
IL-1β	Interleukin-1 Beta
MDD	Major Depressive Disorder
RBANS	Repeatable Battery for the Assessment of Neuropsychological Status
RCTs	Randomized Controlled Trials
SCFAs	Short-Chain Fatty Acids
sIgA	Secretory Immunoglobulin A
WoS	Web of Science

## Appendix A

*Query PubMed:*
(“oral microbiome” OR “oral microbiota” OR “oral dysbiosis” OR “salivary microbiome” OR “dental microbiome”) AND (“gut microbiota” OR “gut microbiome” OR “gut dysbiosis” OR “oral-gut axis” OR “oral-gut microbiota axis”) AND (“gut-brain axis” OR “microbiota-gut-brain axis” OR neuroinflammation OR “systemic inflammation” OR cortisol OR “salivary cortisol” OR “HPA axis”) AND (children OR child OR pediatric OR paediatric OR infant OR infancy OR adolescent OR adolescence OR youth);
*Query Scopus:*
TITLE-ABS-KEY (“oral microbiome” OR “oral dysbiosis”) AND (“gut microbiota” OR “oral-gut axis” OR “oral-gut microbiota axis”) AND (“gut-brain axis” OR “microbiota-gut-brain axis” OR neuroinflammation OR cortisol) AND (children OR pediatric OR paediatric OR infant OR adolescent);
*Query Web of Science:*
TS = (“oral microbiome” OR “oral microbiota” OR “oral dysbiosis” OR “salivary microbiome” OR “dental microbiome”) AND (“gut microbiota” OR “gut microbiome” OR “gut dysbiosis” OR “oral-gut axis” OR “oral-gut microbiota axis”) AND (“gut-brain axis” OR “microbiota-gut-brain axis” OR neuroinflammation OR “systemic inflammation” OR cortisol OR “salivary cortisol” OR “HPA axis”) AND (children OR child OR pediatric OR paediatric OR infant OR infancy OR adolescent OR adolescence OR youth).

## References

[1] Zhang Y., Wang X., Li H., Ni C., Du Z., Yan F. (2018). Human Oral Microbiota and Its Modulation for Oral Health. Biomed. Pharmacother..

[2] Rajasekaran J.J., Krishnamurthy H.K., Bosco J., Jayaraman V., Krishna K., Wang T., Bei K. (2024). Oral Microbiome: A Review of Its Impact on Oral and Systemic Health. Microorganisms.

[3] Glavina A., Martić D., Perko M.A., Mešin Delić D., Tadin A., Lešić S., Šupe-Domić D. (2026). The Oral Microbiome and Systemic Health: Current Insights into the Mouth-Body Connection. Life.

[4] Burcham Z.M., Garneau N.L., Comstock S.S., Tucker R.M., Knight R., Metcalf J.L., Miranda A., Reinhart B., Meyers D., Genetics of Taste Lab Citizen Scientists (2020). Patterns of Oral Microbiota Diversity in Adults and Children: A Crowdsourced Population Study. Sci. Rep..

[5] Milani C., Duranti S., Bottacini F., Casey E., Turroni F., Mahony J., Belzer C., Delgado Palacio S., Arboleya Montes S., Mancabelli L. (2017). The First Microbial Colonizers of the Human Gut: Composition, Activities, and Health Implications of the Infant Gut Microbiota. Microbiol. Mol. Biol. Rev..

[6] Dzidic M., Collado M.C., Abrahamsson T., Artacho A., Stensson M., Jenmalm M.C., Mira A. (2018). Oral Microbiome Development during Childhood: An Ecological Succession Influenced by Postnatal Factors and Associated with Tooth Decay. ISME J..

[7] Kennedy B., Peura S., Hammar U., Vicenzi S., Hedman A., Almqvist C., Andolf E., Pershagen G., Dicksved J., Bertilsson S. (2019). Oral Microbiota Development in Early Childhood. Sci. Rep..

[8] Lif Holgerson P., Harnevik L., Hernell O., Tanner A.C.R., Johansson I. (2011). Mode of Birth Delivery Affects Oral Microbiota in Infants. J. Dent. Res..

[9] Appleton J. (2018). The Gut-Brain Axis: Influence of Microbiota on Mood and Mental Health. Integr. Med..

[10] Azzolino D., Carnevale-Schianca M., Santacroce L., Colella M., Felicetti A., Terranova L., Castrejón-Pérez R.C., Garcia-Godoy F., Lucchi T., Passarelli P.C. (2025). The Oral–Gut Microbiota Axis Across the Lifespan: New Insights on a Forgotten Interaction. Nutrients.

[11] Carabotti M., Scirocco A., Maselli M.A., Severi C. (2015). The Gut-Brain Axis: Interactions between Enteric Microbiota, Central and Enteric Nervous Systems. Ann. Gastroenterol..

[12] Kitamoto S., Nagao-Kitamoto H., Hein R., Schmidt T.M., Kamada N. (2020). The Bacterial Connection between the Oral Cavity and the Gut Diseases. J. Dent. Res..

[13] Cryan J.F., O’Riordan K.J., Cowan C.S.M., Sandhu K.V., Bastiaanssen T.F.S., Boehme M., Codagnone M.G., Cussotto S., Fulling C., Golubeva A.V. (2019). The Microbiota-Gut-Brain Axis. Physiol. Rev..

[14] Schmidt T.S., Hayward M.R., Coelho L.P., Li S.S., Costea P.I., Voigt A.Y., Wirbel J., Maistrenko O.M., Alves R.J., Bergsten E. (2019). Extensive Transmission of Microbes along the Gastrointestinal Tract. eLife.

[15] Reddel S., Pascucci G.R., Foligno S., Del Chierico F., Vernocchi P., Marzullo A., Pattumelli M.G., Palma P., Salvatori G., Putignani L. (2022). A Parallel Tracking of Salivary and Gut Microbiota Profiles Can Reveal Maturation and Interplay of Early Life Microbial Communities in Healthy Infants. Microorganisms.

[16] Lacorte E., Gervasi G., Bacigalupo I., Vanacore N., Raucci U., Parisi P. (2019). A Systematic Review of the Microbiome in Children with Neurodevelopmental Disorders. Front. Neurol..

[17] Li C., Fan Y., Chen X. (2026). Oral Microbiota-Driven Immune Modulation along the Oral-Gut Axis: From Local Signals to Systemic Inflammation. npj Biofilms Microbiomes.

[18] Park S.-Y., Hwang B.-O., Lim M., Ok S.-H., Lee S.-K., Chun K.-S., Park K.-K., Hu Y., Chung W.-Y., Song N.-Y. (2021). Oral-Gut Microbiome Axis in Gastrointestinal Disease and Cancer. Cancers.

[19] Miranda V., Laarej K., Cavaliere C. (2026). The Oral Microbiota: Implications in Mucosal Health and Systemic Disease-Crosstalk with Gut and Brain. Cells.

[20] Xu Q., Wang W., Li Y., Cui J., Zhu M., Liu Y., Liu Y. (2025). The Oral-Gut Microbiota Axis: A Link in Cardiometabolic Diseases. npj Biofilms Microbiomes.

[21] Costa C.F.F.A., Correia-de-Sá T., Araujo R., Barbosa F., Burnet P.W.J., Ferreira-Gomes J., Sampaio-Maia B. (2024). The Oral-Gut Microbiota Relationship in Healthy Humans: Identifying Shared Bacteria between Environments and Age Groups. Front. Microbiol..

[22] Siddiqui Z.R., Rahman S., Verma N., Srivastava A., Jahan N., Mohanty S., Suman S. (2025). Gut–Oral Microbial Dysbiosis: A Correlated Ecosystem. Adv. Gut Microbiome Res..

[23] Beurel E., Nemeroff C.B. (2024). Early Life Adversity, Microbiome, and Inflammatory Responses. Biomolecules.

[24] Mallick R., Basak S., Das R.K., Banerjee A., Paul S., Pathak S., Duttaroy A.K. (2024). Roles of the Gut Microbiota in Human Neurodevelopment and Adult Brain Disorders. Front. Neurosci..

[25] Charitos I.A., Inchingolo A.M., Ferrante L., Inchingolo F., Inchingolo A.D., Castellaneta F., Cotoia A., Palermo A., Scacco S., Dipalma G. (2024). The Gut Microbiota’s Role in Neurological, Psychiatric, and Neurodevelopmental Disorders. Nutrients.

[26] Borrego-Ruiz A., Borrego J.J. (2025). Early-Life Gut Microbiome Development and Its Potential Long-Term Impact on Health Outcomes. Microbiome Res. Rep..

[27] Azzolino D., Carnevale-Schianca M., Bottalico L., Colella M., Felicetti A., Perna S., Terranova L., Garcia-Godoy F., Rondanelli M., Passarelli P.C. (2025). The Oral–Gut Microbiota Axis as a Mediator of Frailty and Sarcopenia. Nutrients.

[28] Jung M., Boutin S., Simon M.M., Frese C. (2025). Comparative Analysis of Oral Microbiome in Molar-Incisor-Hypomineralization vs Healthy Age-Matched Controls. Microbiol. Spectr..

[29] Freire M., Moustafa A., Harkins D.M., Torralba M.G., Zhang Y., Leong P., Saffery R., Bockmann M., Kuelbs C., Hughes T. (2020). Longitudinal Study of Oral Microbiome Variation in Twins. Sci. Rep..

[30] Wang S., Xun Y., Ahern G.J., Feng L., Zhang D., Xue Y., Ross R.P., Doolan A.M., Stanton C., Zhu H. (2021). A Randomized, Double Blind, Parallel, Placebo-Controlled Study to Investigate the Efficacy of Lactobacillus Paracasei N1115 in Gut Development of Young Children. Food Sci. Nutr..

[31] Duarte J.C.M., Costa I.B., Teixeira D.D.B., Fregatto L.F., Mendes C.G., Mascarin A.M.N., Da Silveira Junior S.B., Serva B.E.B.M., Comar L.P., Da Silva R.G. (2023). Biochemical and Microbiological Aspects of the Oral Cavity of Children and Young People with Neurological Impairment and Oropharyngeal Dysphagia. Life.

[32] Xu D., Wan F. (2025). Breastfeeding and Infant Gut Microbiota: Influence of Bioactive Components. Gut Microbes.

[33] Inchingolo F., Inchingolo A.M., Latini G., Ferrante L., De Ruvo E., Campanelli M., Longo M., Palermo A., Inchingolo A.D., Dipalma G. (2024). Difference in the Intestinal Microbiota between Breastfeed Infants and Infants Fed with Artificial Milk: A Systematic Review. Pathogens.

[34] Abbas M., Hayirli Z., Drakesmith H., Andrews S.C., Lewis M.C. (2022). Effects of Iron Deficiency and Iron Supplementation at the Host-Microbiota Interface: Could a Piglet Model Unravel Complexities of the Underlying Mechanisms?. Front. Nutr..

[35] Camacho-Morales A., Caba M., García-Juárez M., Caba-Flores M.D., Viveros-Contreras R., Martínez-Valenzuela C. (2021). Breastfeeding Contributes to Physiological Immune Programming in the Newborn. Front. Pediatr..

[36] Power M.L., Muletz-Wolz C.R., Bornbusch S.L. (2024). Microbiome: Mammalian Milk Microbiomes: Sources of Diversity, Potential Functions, and Future Research Directions. Reprod. Fertil..

[37] Christensen C., Kok C.R., Harris C.L., Moore N., Wampler J.L., Zhuang W., Wu S.S., Hutkins R., Izard J., Auchtung J.M. (2024). Microbiota, Metabolic Profiles and Immune Biomarkers in Infants Receiving Formula with Added Bovine Milk Fat Globule Membrane: A Randomized, Controlled Trial. Front. Nutr..

[38] Corriero A., Gadaleta R.M., Puntillo F., Inchingolo F., Moschetta A., Brienza N. (2022). The Central Role of the Gut in Intensive Care. Crit. Care.

[39] Inchingolo A.D., Marinelli G., Limongelli L., Inchingolo F., Favia G., Ferrante L., Di Noia A., Maspero C., Palermo A., Inchingolo A.M. (2026). Dietary and Complementary Oral Supplements for the Management of Chronic Diseases in Children: A Systematic Review. Front. Pediatr..

[40] Signorini L., Ballini A., Arrigoni R., De Leonardis F., Saini R., Cantore S., De Vito D., Coscia M.F., Dipalma G., Santacroce L. (2021). Evaluation of a Nutraceutical Product with Probiotics, Vitamin D, Plus Banaba Leaf Extracts (Lagerstroemia Speciosa) in Glycemic Control. EMIDDT.

[41] Hasan N., Yang H. (2019). Factors Affecting the Composition of the Gut Microbiota, and Its Modulation. PeerJ.

[42] Sáez-Fuertes L., Kapravelou G., Grases-Pintó B., Bernabeu M., Knipping K., Garssen J., Bourdet-Sicard R., Castell M., Collado M.C., Pérez-Cano F.J. (2024). Maternal Synbiotic Supplementation with B. Breve M-16V and scGOS/lcFOS Shape Offspring Immune Development and Gut Microbiota at the End of Suckling. Nutrients.

[43] Çelik M.N., Dazıroğlu M.E.Ç., Pınar B.A., Nanì M.F., Romano B., Ağagündüz D., Capasso R. (2026). Microbiome Crosstalk and Nutrition: The Interplay between Gut Microbiota-Organ Axis and Dietary Factors. Food Res. Int..

[44] Susic D., Davis G., O’ Sullivan A.J., McGovern E., Harris K., Roberts L.M., Craig M.E., Mangos G., Hold G.L., El-Omar E.M. (2020). Microbiome Understanding in Maternity Study (MUMS), an Australian Prospective Longitudinal Cohort Study of Maternal and Infant Microbiota: Study Protocol. BMJ Open.

[45] Inchingolo A.D., Malcangi G., Semjonova A., Inchingolo A.M., Patano A., Coloccia G., Ceci S., Marinelli G., Di Pede C., Ciocia A.M. (2022). Oralbiotica/Oralbiotics: The Impact of Oral Microbiota on Dental Health and Demineralization: A Systematic Review of the Literature. Children.

[46] Inchingolo F., Inchingolo A.M., Malcangi G., De Leonardis N., Sardano R., Pezzolla C., De Ruvo E., Di Venere D., Palermo A., Inchingolo A.D. (2023). The Benefits of Probiotics on Oral Health: Systematic Review of the Literature. Pharmaceuticals.

[47] Ataei P., Kalantari H., Bodnar T.S., Turner R.J. (2026). The Gut–Brain Connection: Microbes’ Influence on Mental Health and Psychological Disorders. Front. Microbiomes.

[48] Olate P., Martínez A., Sans-Serramitjana E., Cortés M., Díaz R., Hernández G., Paz E.A., Sepúlveda N., Quiñones J. (2025). The Infant Oral Microbiome: Developmental Dynamics, Modulating Factors, and Implications for Oral and Systemic Health. Int. J. Mol. Sci..

[49] Dash S., Syed Y.A., Khan M.R. (2022). Understanding the Role of the Gut Microbiome in Brain Development and Its Association with Neurodevelopmental Psychiatric Disorders. Front. Cell Dev. Biol..

[50] Bevilacqua A., Speranza B., Racioppo A., Santillo A., Albenzio M., Derossi A., Caporizzi R., Francavilla M., Racca D., Flagella Z. (2024). Ultra-Processed Food and Gut Microbiota: Do Additives Affect Eubiosis? A Narrative Review. Nutrients.

[51] Shi Z. (2019). Gut Microbiota: An Important Link between Western Diet and Chronic Diseases. Nutrients.

[52] Sakagianni A., Koufopoulou C., Koufopoulos P., Feretzakis G., Anastasiou A., Theodorakis N., Myrianthefs P. (2025). Influence of Microbiome Interactions on Antibiotic Resistance Development in the ICU Environment: Insights and Opportunities with Machine Learning. Acta Microbiol. Hell..

[53] Inchingolo F., Inchingolo A.D., Palumbo I., Trilli I., Guglielmo M., Mancini A., Palermo A., Inchingolo A.M., Dipalma G. (2024). The Impact of Cesarean Section Delivery on Intestinal Microbiota: Mechanisms, Consequences, and Perspectives—A Systematic Review. Int. J. Mol. Sci..

[54] Macrì F., Dipalma G., Inchingolo A.M., Inchingolo F., Maspero C., Giannini L. (2026). Breastfeeding vs. Formula Feeding on Maternal Oral Health: Periodontal and Microbiological Changes in the Postpartum Period: An Observational Longitudinal Study. Front. Oral Health.

[55] Davis E.C., Castagna V.P., Sela D.A., Hillard M.A., Lindberg S., Mantis N.J., Seppo A.E., Järvinen K.M. (2022). Gut Microbiome and Breast-Feeding: Implications for Early Immune Development. J. Allergy Clin. Immunol..

[56] Ramadugu K., Bhaumik D., Luo T., Gicquelais R.E., Lee K.H., Stafford E.B., Marrs C.F., Neiswanger K., McNeil D.W., Marazita M.L. (2021). Maternal Oral Health Influences Infant Salivary Microbiome. J. Dent. Res..

[57] Zhang Y., Wu Y.-P., Feng V., Cao G.-Z., Feng X.-P., Chen X. (2022). Microbiota of Preterm Infant Develops over Time along with the First Teeth Eruption. Front. Microbiol..

[58] Laforest-Lapointe I., Arrieta M.-C. (2017). Patterns of Early-Life Gut Microbial Colonization during Human Immune Development: An Ecological Perspective. Front. Immunol..

[59] Ciulla M.M., Re D., Gilardoni E., D’Amato A., Altomare A., Baron G., Carugo S., Aldini G. (2021). PHoral: Effects of Carnosine Supplementation on Quantity/Quality of Oral Salivae in Healthy Volunteer and in Subjects Affected by Common Oral Pathologies. Medicine.

[60] Coker M.O., Lebeaux R.M., Hoen A.G., Moroishi Y., Gilbert-Diamond D., Dade E.F., Palys T.J., Madan J.C., Karagas M.R. (2022). Metagenomic Analysis Reveals Associations between Salivary Microbiota and Body Composition in Early Childhood. Sci. Rep..

[61] Malcangi G., Patano A., Ciocia A.M., Netti A., Viapiano F., Palumbo I., Trilli I., Guglielmo M., Inchingolo A.D., Dipalma G. (2023). Benefits of Natural Antioxidants on Oral Health. Antioxidants.

[62] Lee E., Park S., Um S., Kim S., Lee J., Jang J., Jeong H., Shin J., Kang J., Lee S. (2021). Microbiome of Saliva and Plaque in Children According to Age and Dental Caries Experience. Diagnostics.

[63] Bull-Larsen S., Mohajeri M.H. (2019). The Potential Influence of the Bacterial Microbiome on the Development and Progression of ADHD. Nutrients.

[64] Yao X., Nie W., Chen X., Zhang J., Wei J., Qiu Y., Liu K., Shao D., Liu H., Ma Z. (2024). Two Enterococcus Faecium Isolates Demonstrated Modulating Effects on the Dysbiosis of Mice Gut Microbiota Induced by Antibiotic Treatment. Int. J. Mol. Sci..

[65] Agustin T.P., Sutadi H., Bachtiar B.M., Rizal M.F. (2024). Proportion of Streptococcus Mutans, Streptococcus Sanguinis, and Candida Albicans in Early Childhood Caries: Evaluation by qPCR. Open Dent. J..

[66] Staszczyk M., Jamka-Kasprzyk M., Kościelniak D., Cienkosz-Stepańczak B., Krzyściak W., Jurczak A. (2022). Effect of a Short-Term Intervention with Lactobacillus Salivarius Probiotic on Early Childhood Caries—An Open Label Randomized Controlled Trial. Int. J. Environ. Res. Public Health.

[67] Grier A., Myers J.A., O’Connor T.G., Quivey R.G., Gill S.R., Kopycka-Kedzierawski D.T. (2021). Oral Microbiota Composition Predicts Early Childhood Caries Onset. J. Dent. Res..

[68] Brotman R.M. (2011). Vaginal Microbiome and Sexually Transmitted Infections: An Epidemiologic Perspective. J. Clin. Investig..

[69] Tynior W., Kłósek M., Salatino S., Cuber P., Hudy D., Nałęcz D., Chan Y.-T., Gustave C., Strzelczyk J.K. (2024). Metagenomic Analysis of the Buccal Microbiome by Nanopore Sequencing Reveals Structural Differences in the Microbiome of a Patient with Molar Incisor Hypomineralization (MIH) Compared to a Healthy Child—Case Study. Int. J. Mol. Sci..

[70] Wejryd E., Marchini G., Bang P., Jonsson B., Ådén U., Abrahamsson T. (2025). Neurodevelopment and Growth 2 Years After Probiotic Supplementation in Extremely Preterm Infants: A Randomised Trial. Acta Paediatr..

[71] Teanpaisan R., Surachat K., Wonglapsuwan M., Piwat S., Pahumunto N. (2024). Short-term Use of *Lacticaseibacillus Rhamnosus*
SD11 and the Oral Microbiome: Low Caries RCT Study. Oral Dis..

[72] Inquimbert C., Bourgeois D., Bravo M., Viennot S., Tramini P., Llodra J.C., Molinari N., Dussart C., Giraudeau N., Carrouel F. (2019). The Oral Bacterial Microbiome of Interdental Surfaces in Adolescents According to Carious Risk. Microorganisms.

[73] Xu X., Shan B., Zhang Q., Lu W., Zhao J., Zhang H., Chen W. (2023). Oral Microbiome Characteristics in Children with and without Early Childhood Caries. J. Clin. Pediatr. Dent..

[74] Deng Z.-L., Szafrański S.P., Jarek M., Bhuju S., Wagner-Döbler I. (2017). Dysbiosis in Chronic Periodontitis: Key Microbial Players and Interactions with the Human Host. Sci. Rep..

[75] Abusleme L., Dupuy A.K., Dutzan N., Silva N., Burleson J.A., Strausbaugh L.D., Gamonal J., Diaz P.I. (2013). The Subgingival Microbiome in Health and Periodontitis and Its Relationship with Community Biomass and Inflammation. ISME J..

[76] Pappa E., Vastardis H., Makridakis M., Zoidakis J., Vougas K., Stamatakis G., Samiotaki M., Rahiotis C. (2022). Analysis of Human and Microbial Salivary Proteomes in Children Offers Insights on the Molecular Pathogenesis of Molar-Incisor Hypomineralization. Biomedicines.

[77] Inchingolo A.D., Malcangi G., Inchingolo A.M., Piras F., Settanni V., Garofoli G., Palmieri G., Ceci S., Patano A., De Leonardis N. (2022). Benefits and Implications of Resveratrol Supplementation on Microbiota Modulations: A Systematic Review of the Literature. Int. J. Mol. Sci..

[78] Shen Y., Fan N., Ma S.-X., Cheng X., Yang X., Wang G. (2025). Gut Microbiota Dysbiosis: Pathogenesis, Diseases, Prevention, and Therapy. MedComm.

[79] Antoniadou M., Varzakas T. (2026). Oral Cellular Homeostasis and Occupational Wellbeing in Healthcare Professionals Under the Lens of Salivary, Immune, and Microbiome Mechanisms. Cells.

[80] Isacco C.G., Ballini A., De Vito D., Nguyen K.C.D., Cantore S., Bottalico L., Quagliuolo L., Boccellino M., Di Domenico M., Santacroce L. (2021). Rebalancing the Oral Microbiota as an Efficient Tool in Endocrine, Metabolic and Immune Disorders. Endocr. Metab. Immune Disord.-Drug Targets.

[81] Inchingolo F., Inchingolo A.D., Latini G., Trilli I., Ferrante L., Nardelli P., Malcangi G., Inchingolo A.M., Mancini A., Palermo A. (2024). The Role of Curcumin in Oral Health and Diseases: A Systematic Review. Antioxidants.

[82] Elzayat H., Mesto G., Al-Marzooq F. (2023). Unraveling the Impact of Gut and Oral Microbiome on Gut Health in Inflammatory Bowel Diseases. Nutrients.

[83] Maki K.A., Kazmi N., Barb J.J., Ames N. (2021). The Oral and Gut Bacterial Microbiomes: Similarities, Differences, and Connections. Biol. Res. Nurs..

[84] Li X., Liu Y., Yang X., Li C., Song Z. (2022). The Oral Microbiota: Community Composition, Influencing Factors, Pathogenesis, and Interventions. Front. Microbiol..

[85] Chen X., Wang N., Wang J., Liao B., Cheng L., Ren B. (2022). The Interactions between Oral-Gut Axis Microbiota and Helicobacter Pylori. Front. Cell. Infect. Microbiol..

[86] Bankole T., Li Y. (2025). The Early-Life Gut Microbiome in Common Pediatric Diseases: Roles and Therapeutic Implications. Front. Nutr..

[87] Nakajima M., Arimatsu K., Kato T., Matsuda Y., Minagawa T., Takahashi N., Ohno H., Yamazaki K. (2015). Oral Administration of P. Gingivalis Induces Dysbiosis of Gut Microbiota and Impaired Barrier Function Leading to Dissemination of Enterobacteria to the Liver. PLoS ONE.

[88] Arimatsu K., Yamada H., Miyazawa H., Minagawa T., Nakajima M., Ryder M.I., Gotoh K., Motooka D., Nakamura S., Iida T. (2014). Oral Pathobiont Induces Systemic Inflammation and Metabolic Changes Associated with Alteration of Gut Microbiota. Sci. Rep..

[89] Harrandah A.M. (2025). The Oral-Gut-Systemic Axis: Emerging Insights into Periodontitis, Microbiota Dysbiosis, and Systemic Disease Interplay. Diagnostics.

[90] Rodríguez G., Ruiz B., Faleiros S., Vistoso A., Marró M.L., Sánchez J., Urzúa I., Cabello R. (2016). Probiotic Compared with Standard Milk for High-Caries Children: A Cluster Randomized Trial. J. Dent. Res..

[91] Piwat S., Teanpaisan R., Manmontri C., Wattanarat O., Pahumunto N., Makeudom A., Krisanaprakornkit S., Nirunsittirat A. (2020). Efficacy of Probiotic Milk for Caries Regression in Preschool Children: A Multicenter Randomized Controlled Trial. Caries Res..

[92] Wattanarat O., Nirunsittirat A., Piwat S., Manmontri C., Teanpaisan R., Pahumunto N., Makeudom A., Sastraruji T., Krisanaprakornkit S. (2021). Significant Elevation of Salivary Human Neutrophil Peptides 1-3 Levels by Probiotic Milk in Preschool Children with Severe Early Childhood Caries: A Randomized Controlled Trial. Clin. Oral Investig..

[93] Salim H.P., Mallikarjun S.B., Raju S., Surendranath A.R. (2023). Randomized Clinical Trial of Oral Probiotic Streptococcus Salivarius M18 on Salivary Streptococcus Mutans in Preprimary Children. Int. J. Clin. Pediatr. Dent..

[94] Sakaryalı Uyar D., Üsküdar Güçlü A., Çelik E., Memiş Özgül B., Altay Koçak A., Başustaoğlu A.C. (2024). Evaluation of Probiotics’ Efficiency on Cariogenic Bacteria: Randomized Controlled Clinical Study. BMC Oral Health.

[95] Tanaka M., Nakayama J. (2017). Development of the Gut Microbiota in Infancy and Its Impact on Health in Later Life. Allergol. Int..

[96] Zhao M., Chu J., Feng S., Guo C., Xue B., He K., Li L. (2023). Immunological Mechanisms of Inflammatory Diseases Caused by Gut Microbiota Dysbiosis: A Review. Biomed. Pharmacother..

[97] Ghosh S., Whitley C.S., Haribabu B., Jala V.R. (2021). Regulation of Intestinal Barrier Function by Microbial Metabolites. Cell. Mol. Gastroenterol. Hepatol..

[98] DuPont H.L. (2014). Review Article: Evidence for the Role of Gut Microbiota in Irritable Bowel Syndrome and Its Potential Influence on Therapeutic Targets. Aliment. Pharmacol. Ther..

[99] Cani P.D., Bibiloni R., Knauf C., Waget A., Neyrinck A.M., Delzenne N.M., Burcelin R. (2008). Changes in Gut Microbiota Control Metabolic Endotoxemia-Induced Inflammation in High-Fat Diet–Induced Obesity and Diabetes in Mice. Diabetes.

[100] Chelakkot C., Ghim J., Ryu S.H. (2018). Mechanisms Regulating Intestinal Barrier Integrity and Its Pathological Implications. Exp. Mol. Med..

[101] Wang H.-B., Wang P.-Y., Wang X., Wan Y.-L., Liu Y.-C. (2012). Butyrate Enhances Intestinal Epithelial Barrier Function via Up-Regulation of Tight Junction Protein Claudin-1 Transcription. Dig. Dis. Sci..

[102] Peng L., Li Z.-R., Green R.S., Holzmanr I.R., Lin J. (2009). Butyrate Enhances the Intestinal Barrier by Facilitating Tight Junction Assembly via Activation of AMP-Activated Protein Kinase in Caco-2 Cell Monolayers. J. Nutr..

[103] Herman J.P., McKlveen J.M., Ghosal S., Kopp B., Wulsin A., Makinson R., Scheimann J., Myers B., Prakash Y.S. (2016). Regulation of the Hypothalamic-Pituitary-Adrenocortical Stress Response. Comprehensive Physiology.

[104] Ballini A., Dipalma G., Isacco C.G., Boccellino M., Di Domenico M., Santacroce L., Nguyễn K.C.D., Scacco S., Calvani M., Boddi A. (2020). Oral Microbiota and Immune System Crosstalk: A Translational Research. Biology.

[105] Shi S., Zhang S., Kong L. (2023). Effects of Treatment with Probiotics on Cognitive Function and Regulatory Role of Cortisol and IL-1β in Adolescent Patients with Major Depressive Disorder. Life.

[106] Calderón-Garcidueñas L., Solt A.C., Henríquez-Roldán C., Torres-Jardón R., Nuse B., Herritt L., Villarreal-Calderón R., Osnaya N., Stone I., García R. (2008). Long-Term Air Pollution Exposure Is Associated with Neuroinflammation, an Altered Innate Immune Response, Disruption of the Blood-Brain Barrier, Ultrafine Particulate Deposition, and Accumulation of Amyloid β-42 and α-Synuclein in Children and Young Adults. Toxicol. Pathol..

[107] Inchingolo F., Inchingolo A.M., Piras F., Ferrante L., Mancini A., Palermo A., Inchingolo A.D., Dipalma G. (2024). The Interaction between Gut Microbiome and Bone Health. Curr. Opin. Endocrinol. Diabetes Obes..

[108] Xie L., Lin W. (2025). The Role of Gut Microbiota Dysbiosis in the Inflammatory Pathogenesis of Diabetic Retinopathy. Front Immunol..

[109] Li J., Cai H., Zhang Y., Li J., Wang D., Li H., Cai H., Wang Q., Fu T., Shao Z. (2024). Dysbiosis of Gut Microbiota Is Associated with Pathogenesis of Peptic Ulcer Diseases through Inflammatory Proteins: A Mendelian Randomization Study. Medicine.

[110] Kulp A., Kuehn M.J. (2010). Biological Functions and Biogenesis of Secreted Bacterial Outer Membrane Vesicles. Annu. Rev. Microbiol..

[111] Mulet M., Sánchez Milán J.A., Lorca C., Fernández-Rhodes M., Adrados-Planell A., Bejarano Castillo M.C., Saiz L., Mateos-Moreno M.-V., Hase Y., Mira A. (2025). Oral Microbiome-Derived Proteins in Brain Extracellular Vesicles Circulate and Tie to Specific Dysbiotic and Neuropathological Profiles in Age-Related Dementias. Mol. Cell Proteom..

[112] Marano G., Sfratta G., Marzo E.M., Cozzo G., Abate F., Traversi G., Mazza O., Capristo E., Gaetani E., Mazza M. (2025). The Pediatric Microbiota-Gut-Brain Axis: Implications for Neuropsychiatric Development and Intervention. Children.

[113] Ellis T.N., Kuehn M.J. (2010). Virulence and Immunomodulatory Roles of Bacterial Outer Membrane Vesicles. Microbiol. Mol. Biol. Rev..

[114] Saeed N.K., Elbeltagi Y.M., Al-Beltagi M. (2026). Unveiling the Viral Dimension: The Paediatric Gut Virome as a Key Modulator of Gastrointestinal Metabolic, and Neurodevelopmental Health. World J. Virol..

[115] Li P., Ren Z., Zhou J., Zhao A., Wang S., Xun Y., Jiang H., Wang P., Yuan Q., Zhang Y. (2023). Effect of Lacticaseibacillus Paracasei N1115 on Immunomodulatory and Gut Microbial Composition in Young Children: A Randomized, Placebo-Controlled Study. Nutrients.

[116] Xie Z., Chen Z., Chai Y., Yao W., Ma G. (2025). Unveiling the Placental Bacterial Microbiota: Implications for Maternal and Infant Health. Front. Physiol..

[117] Dipalma G., Marinelli G., Ferrante L., Di Noia A., Carone C., Colonna V., Marotti P., Inchingolo F., Palermo A., Tartaglia G.M. (2025). Modulating the Gut Microbiota to Target Neuroinflammation, Cognition and Mood: A Systematic Review of Human Studies with Relevance to Fibromyalgia. Nutrients.

[118] Inchingolo A.M., Patano A., Piras F., Mancini A., Inchingolo A.D., Paduanelli G., Inchingolo F., Palermo A., Dipalma G., Malcangi G. (2023). Interconnection between Microbiota–Gut–Brain Axis and Autism Spectrum Disorder Comparing Therapeutic Options: A Scoping Review. Microorganisms.

[119] Bravo J.A., Forsythe P., Chew M.V., Escaravage E., Savignac H.M., Dinan T.G., Bienenstock J., Cryan J.F. (2011). Ingestion of Lactobacillus Strain Regulates Emotional Behavior and Central GABA Receptor Expression in a Mouse via the Vagus Nerve. Proc. Natl. Acad. Sci. USA.

[120] Brett B.E., Koko B.K., Doumbia H.O.Y., Koffi F.K., Assa S.E., Zahé K.Y.A.S., Faye-Ketté H., Kati-Coulibaly S., Kort R., Sybesma W. (2021). Salivary Biomarkers of Stress and Inflammation in First Graders in Côte d′Ivoire: Effects of a Probiotic Food Intervention. Psychoneuroendocrinology.

[121] Foster J.A., Rinaman L., Cryan J.F. (2017). Stress & the Gut-Brain Axis: Regulation by the Microbiome. Neurobiol. Stress.

[122] Inchingolo F., Inchingolo A.M., Avantario P., Settanni V., Fatone M.C., Piras F., Di Venere D., Inchingolo A.D., Palermo A., Dipalma G. (2023). The Effects of Periodontal Treatment on Rheumatoid Arthritis and of Anti-Rheumatic Drugs on Periodontitis: A Systematic Review. Int. J. Mol. Sci..

[123] Braniste V., Al-Asmakh M., Kowal C., Anuar F., Abbaspour A., Tóth M., Korecka A., Bakocevic N., Ng L.G., Kundu P. (2014). The Gut Microbiota Influences Blood-Brain Barrier Permeability in Mice. Sci. Transl. Med..

[124] Feng Y.-K., Wu Q.-L., Peng Y.-W., Liang F.-Y., You H.-J., Feng Y.-W., Li G., Li X.-J., Liu S.-H., Li Y.-C. (2020). Oral P. Gingivalis Impairs Gut Permeability and Mediates Immune Responses Associated with Neurodegeneration in LRRK2 R1441G Mice. J. Neuroinflammation.

[125] Sheng J.A., Bales N.J., Myers S.A., Bautista A.I., Roueinfar M., Hale T.M., Handa R.J. (2020). The Hypothalamic-Pituitary-Adrenal Axis: Development, Programming Actions of Hormones, and Maternal-Fetal Interactions. Front. Behav. Neurosci..

[126] Yassin L.K., Skrabulyte-Barbulescu J., Alshamsi S.H., Saeed S., Alkuwaiti S.H., Almazrouei S., Alnuaimi A., BaniYas S., Aldhaheri D., Alderei M. (2025). The Microbiota-Gut-Brain Axis in Mental and Neurodegenerative Disorders: Opportunities for Prevention and Intervention. Front Aging Neurosci..

[127] Ring M. (2025). An Integrative Approach to HPA Axis Dysfunction: From Recognition to Recovery. Am. J. Med..

[128] Fossati A.L., Sobral A.P.T., Hermida Bruno M.L.L., Viarengo N.O., Sertaje M.R.F., Santos E.M., Gonçalves M.L.L., Ferrari R.A.M., Fernandes K.P.S., Horliana A.C.R.T. (2023). Photobiomodulation and Glass Ionomer Sealant as Complementary Treatment for Hypersensitivity in Molar Incisor Hypomineralisation in Children: Protocol for a Blinded Randomised Clinical Trial. BMJ Open.

[129] Sudo N., Chida Y., Aiba Y., Sonoda J., Oyama N., Yu X.-N., Kubo C., Koga Y. (2004). Postnatal Microbial Colonization Programs the Hypothalamic-Pituitary-Adrenal System for Stress Response in Mice. J. Physiol..

[130] Radaic A., Kapila Y.L. (2021). The Oralome and Its Dysbiosis: New Insights into Oral Microbiome-Host Interactions. Comput. Struct. Biotechnol. J..

[131] Xiang Z., Liu Y. (2026). The Pediatric Oral Mycobiome: A Comprehensive Review of Its Role in Health and Disease. Front. Cell. Infect. Microbiol..

[132] Adam E.K., Kumari M. (2009). Assessing Salivary Cortisol in Large-Scale, Epidemiological Research. Psychoneuroendocrinology.

[133] Keil M.F. (2012). Salivary Cortisol: A Tool for Biobehavioral Research in Children. J. Pediatr. Nurs..

[134] Strahler J., Skoluda N., Kappert M.B., Nater U.M. (2017). Simultaneous Measurement of Salivary Cortisol and Alpha-Amylase: Application and Recommendations. Neurosci. Biobehav. Rev..

[135] Garde A.H., Persson R., Hansen Å.M., Österberg K., Ørbæk P., Eek F., Karlson B. (2009). Effects of Lifestyle Factors on Concentrations of Salivary Cortisol in Healthy Individuals. Scand. J. Clin. Lab. Investig..

[136] Dong F., Sefcik J.S., Euiler E., Hodgson N.A. (2025). Measuring Salivary Cortisol in Biobehavioral Research: A Systematic Review and Methodological Considerations. Brain Behav. Immun.-Health.

[137] Herrera-Quintana L., Vázquez-Lorente H., Hinojosa-Nogueira D., Plaza-Diaz J. (2024). Relationship between Infant Feeding and the Microbiome: Implications for Allergies and Food Intolerances. Children.

[138] Perrett B.M., Miliku K., Moraes T.J., Simons E., Mandhane P., Kebbe M. (2026). Gut Microbiota Responses to Complementary Food Sources Differ by Milk Feeding Type. Clin. Nutr..

[139] Calderón-Garcidueñas L., Franco-Lira M., D’Angiulli A., Rodríguez-Díaz J., Blaurock-Busch E., Busch Y., Chao C., Thompson C., Mukherjee P.S., Torres-Jardón R. (2015). Mexico City Normal Weight Children Exposed to High Concentrations of Ambient PM2.5 Show High Blood Leptin and Endothelin-1, Vitamin D Deficiency, and Food Reward Hormone Dysregulation versus Low Pollution Controls. Relevance for Obesity and Alzheimer Disease. Environ. Res..

[140] Albalooshy A. (2024). Vitamin D Deficiency and Chronological Hypoplasia with Hypomineralisation: A Case Report. J. Clin. Pediatr. Dent..

[141] Sakkas H., Bozidis P., Touzios C., Kolios D., Athanasiou G., Athanasopoulou E., Gerou I., Gartzonika C. (2020). Nutritional Status and the Influence of the Vegan Diet on the Gut Microbiota and Human Health. Medicina.

[142] Hansen T.H., Kern T., Bak E.G., Kashani A., Allin K.H., Nielsen T., Hansen T., Pedersen O. (2018). Impact of a Vegan Diet on the Human Salivary Microbiota. Sci. Rep..

[143] Perrone P., D’Angelo S. (2025). Gut Microbiota Modulation Through Mediterranean Diet Foods: Implications for Human Health. Nutrients.

[144] Haskey N., Estaki M., Ye J., Shim R.K., Singh S., Dieleman L.A., Jacobson K., Gibson D.L. (2023). A Mediterranean Diet Pattern Improves Intestinal Inflammation Concomitant with Reshaping of the Bacteriome in Ulcerative Colitis: A Randomised Controlled Trial. J. Crohn’s Colitis.

[145] Sepehrimanesh M., Melen S.V., Yeasmin F., Ojo V.A., Walden F., Urmee H., Etheridge J., Nasu A.K. (2026). Emerging Therapeutic Strategies for Neurodegenerative Diseases: A Comprehensive Review of Recent Advances and Future Directions. Cells.

[146] Olczak-Kowalczyk D., Turska-Szybka A., Twetman S., Gozdowski D., Piekoszewska-Ziętek P., Góra J., Wróblewska M. (2024). Effect of Tablets Containing a Paraprobiotic Strain and the Cranberry Extract on Caries Incidence in Preschool Children: A Randomized Controlled Trial. Dent. Med. Probl..

[147] Huang Y., Liang Q., Shen Y., Chen J., Xu W. (2026). Oral Microbiome Dysbiosis in Autism Spectrum Disorder: The Oral-Gut-Brain Axis and Future Perspectives: A Narrative Review. Front Microbiol..

[148] Dumitru C.N., Dumitru A.O., Popa G.V., Marcu T., Ursu M., Nechita A., Matei N.M. (2026). The Oral-Gut-Brain Axis: From Periodontal Dysbiosis to Neuroinflammation-Mechanistic Pathways, Salivary and Intestinal Biomarkers, and Therapeutic Targets: A Narrative Review. Dent. J..

[149] Zhang J.S., Chu C.-H., Yu O.Y. (2022). Oral Microbiome and Dental Caries Development. Dent. J..

[150] Cowan C.S.M., Dinan T.G., Cryan J.F. (2020). Annual Research Review: Critical Windows—The Microbiota–Gut–Brain Axis in Neurocognitive Development. Child. Psychol. Psychiatry.

[151] Jadhav G., Hegde R., Jadhav Y.J., Shigli A., Herkar P. (2024). Gut-Brain-Oral Dysbiosis: A Comprehensive Review. Contemp. Pediatr. Dent..

[152] Adil N.A., Omo-Erigbe C., Yadav H., Jain S. (2025). The Oral–Gut Microbiome–Brain Axis in Cognition. Microorganisms.

[153] Brandon N.J., Millar J.K., Korth C., Sive H., Singh K.K., Sawa A. (2009). Understanding the Role of DISC1 in Psychiatric Disease and during Normal Development. J. Neurosci..

